# Effects of pressure-controlled ventilation-volume guaranteed on children undergoing thoracic surgery: a prospective, randomized controlled trial

**DOI:** 10.3389/fmed.2025.1647682

**Published:** 2025-09-15

**Authors:** Lei Wang, Yi Zhu, Shili Zhu, Lulu Yan, Zhen Du, Ting Xiao, Shuangquan Qu

**Affiliations:** Department of Anesthesiology, The Affiliated Children's Hospital of Xiangya School of Medicine, Central South University (Hunan Children's Hospital), Changsha, China

**Keywords:** pressure-controlled ventilation-volume guaranteed, one-lung ventilation, children, intrapulmonary shunt, video-assisted thoracoscopic surgery, ventilator-induced lung injury

## Abstract

**Background:**

Children have higher morbidity and mortality than adults during thoracic surgery with one-lung ventilation (OLV), reducing the incidence may in part be achieved by the use of appropriate mechanical ventilation mode. This study evaluated whether pressure-controlled ventilation-volume guaranteed (PCV-VG) reduces intrapulmonary shunt (Qs/Qt) and ventilator-induced lung injury (VILI) in pediatric patients.

**Methods:**

Eighty children underwent thoracic surgery requiring OLV were randomly divided into PCV-VG group and volume-controlled ventilation (VCV) group. The PCV-VG group utilized the PCV-VG during surgery, whereas the other group employed the VCV. The parameter settings during two-lung and OLV were consistent between the groups. The primary outcome comprised the Qs/Qt fraction. The secondary outcomes encompassed respiratory parameters, blood gas analysis results, the occurrence of postoperative pulmonary complications (PPCs), and more.

**Results:**

Following PCV-VG implementation, the median Qs/Qt during OLV exhibited a significant improvement (18.2 to 12.7 at T2, 11.4 to 9.1 at T3, *p* < 0.0001). Additionally, compared with VCV, PCV-VG improved oxygenation during OLV (higher PaO_2_, PaO_2_/FiO_2_, and other oxygenation indices), reduced airway pressure during OLV (25cmH_2_O to 20cmH_2_O at T2, *p* < 0.0001), and lowered the incidence of PPCs [38.5 to 7.3%, adjusted odds ratio (OR) 0.13, 95% confidence interval (CI): 0.03–0.50, *p* = 0.001], shortened the duration of postoperative mechanical ventilation [120 min to 110 min, difference (95% CI), 20 (0 to 40), *p* = 0.036].

**Conclusion:**

Implementation of PCV-VG for children undergoing thoracic surgery demonstrated significant benefits. It can improve Qs/Qt, increase oxygenation, reduce airway pressure, and alleviate VILI during OLV.

**Clinical trial registration:**

Clinicaltrials.gov, identifier ChiCTR2200065237.

## Highlights

- Optimal ventilation modes may reduce morbidity/mortality in Children undergoing thoracic surgery with one-lung ventilation, though the optimal strategy remains uncertain.
- PCV-VG can improve intrapulmonary shunting, enhances oxygenation, mitigate ventilator-induced lung injury, and improves outcomes in pediatric thoracic surgery.
- This trial firstly confirms that PCV-VG is a more beneficial ventilation method during one-lung ventilation in pediatric thoracic surgery.

## 1 Introduction

One-lung ventilation (OLV) provides a clear surgical field of vision and protects the healthy lung from contamination, making it an essential technique in video-assisted thoracic surgery (1). Nevertheless, OLV in video-assisted thoracoscopic surgery adversely affects the respiratory system, leading to an imbalance in ventilation/blood flow ratio, increased intrapulmonary shunt (Qs/Qt) fraction, and decreased partial arterial oxygen pressure (PaO_2_) (2, 3). Children have higher morbidity and mortality rates during thoracic surgery requiring OLV due to their smaller functional residual capacity, larger closing volume, and greater chest wall compliance (4, 5). Reducing the incidence above and improving clinical outcomes can be partly achieved by utilizing appropriate mechanical ventilation mode (6–8).

Modern anesthesia machines perform well in accurately delivering the required volume and pressure through various ventilation modes. Volume-controlled ventilation (VCV) is most commonly used in pediatric thoracic surgery (5, 9), providing stable ventilation and reducing the incidence of postoperative pulmonary complications (PPCs) (10, 11). Nevertheless, some studies suggest that VCV may increase the release of inflammatory mediators, peak inspiratory pressure, and worsen dynamic compliance in thoracic surgical patients (12, 13). Pressure-controlled ventilation-volume guaranteed (PCV-VG) combines the benefits of pressure-controlled ventilation and VCV. It can not only maintain the set minute ventilation, but also reduce incidence of barotrauma (14). The benefits of applying PCV-VG on clinical outcomes among adult surgical patients have been confirmed in many trials (2, 15, 16). However, unlike adults, the types of diseases that necessitate thoracic surgery in children are mostly congenital malformations with mild symptoms and better lung conditions. Therefore, it remains unclear whether PCV-VG offers comparable benefits in pediatric OLV.

Investigations into the optimal ventilation mode during pediatric OLV are essential and will contribute valuable information to the best practices in this field. Hence, we initiated this prospective randomized controlled trial (RCT) to assess the impact of using PCV-VG mode in children undergoing video-assisted thoracoscopic lobectomy with OLV on intrapulmonary shunt, oxygenation, respiratory mechanics, and other clinical outcomes. The hypothesis posits that the application of PCV-VG during pediatric OLV can enhance intraoperative Qs/Qt, improve thoracopulmonary compliance and perioperative oxygenation, decrease airway pressure, and reduce the incidence of VILI.

## 2 Methods

### 2.1 Ethics statement

The study was performed in accordance with the ethical principles outlined in the Declaration of Helsinki. Ethical approval for this study (approval number: HCHLL-2022-92) was provided by the institutional ethics committee of Hunan Children’s Hospital, China (Chairperson Prof Zhenghui Xiao) on 23 September 2022. Then this study was registered at the Chinese Clinical Trial Registry (ChiCTR2200065237, registry URL: www.chictr.org.cn) before implementation. Prior to randomization and surgery, the researcher elucidated the study protocol to both the child and their guardians, securing written informed consent from the guardians.

### 2.2 Inclusion and exclusion criteria

Eligibility assessment for patients took place on the day before their surgery. Inclusion criteria covered patients aged 6 months to 18 years, falling within American Society of Anesthesiologists (ASA) classes I - III, and undergoing video-assisted thoracoscopic lobectomy with OLV. Exclusion criteria comprised patients with ongoing respiratory infections and asthma prior to surgery, severe organ dysfunction (such as severe liver and kidney dysfunction, congenital heart disease), or individuals unwilling to participate in the study.

### 2.3 Group allocation

Prior to enrollment, 132 children scheduled for elective video-assisted thoracoscopic lobectomy were screened for eligibility. Ultimately, 83 children were enrolled in the study. Eligible children were randomly assigned to either group in a 1:1 ratio using a computer-generated allocation scheme to ensure group homogeneity. Participants were randomly assigned in advance to either the PCV-VG group or the VCV group. Forty-one children were allocated to the VCV group, whereas 42 children to the PCV-VG group. A study coordinator conducted follow-ups on each enrolled patient to ensure rigorous adherence to the trial protocol. The allocation scheme was concealed in sequentially numbered opaque envelopes. The anesthetists in charge were aware of the allocation group, while the researchers collecting the data, surgical team, and nursing staff remained blinded to the assignment.

### 2.4 Anesthesia induction and maintenance

Throughout the anesthesia, continuous monitoring was conducted for pulse oximetry, electrocardiography, bispectral index (BIS), and invasive arterial blood pressure. Intravenous induction of anesthesia was performed using esketamine (0.5–1 mg.kg-1), cisatracurium (0.2 mg.kg-1), propofol (2–3 mg.kg-1), and sufentanil (0.3–0.5 ug.kg-1). Following induction, an endotracheal tube of suitable size and a bronchial blocker (Arndt blocker; Cook Critical Care, Bloomington, IL, USA) of size 5 Fr or 7 Fr, determined by the main bronchus size on the operative side, were inserted. Fiberoptic bronchoscopy (Olympus LF-DP; Olympus Corporation, Tokyo, Japan) was utilized to assist in the introduction of the bronchial blocker. Once the position of the bronchial blocker was confirmed, the endotracheal tube was connected to the identical mechanical ventilator (A7; Mindary, Guangdong, China) for mechanical ventilation in both groups. Following the placement of endotracheal tube and bronchial blocker, anesthesia was maintained with sevoflurane (≤ 1 minimal alveolar concentration) and a continuous infusion of propofol (3–5 mg.kg.h-1), remifentanil (0.2–0.5 ug.kg.min-1) and dexmedetomidine (0.2–0.5 ug.kg.h-1) to maintain a BIS value of 40 to 60. Intermittent intravenous injection of 0.1 mg/kg cisatracurium.

All lobectomies were performed by the same group of surgeons using video-assisted thoracoscopy. In terms of fluid management, both groups received uniform management to avoid excessive fluid administration.

After surgery, each child was transferred to the cardiothoracic surgical care unit while still intubated. The postoperative ventilators used by the two groups of children were the same as those used during surgery, and the ventilation mode was the same as their original grouping (VCV *vs.* PCV-VG). The respiratory parameters were set the same as for two-lung ventilation (TLV). Extubation was carried out when predefined criteria were met, including a regular respiratory rhythm, adequate recovery of muscle strength, presence of respiratory protective reflexes, and absence of factors contributing to airway obstruction. The decision to proceed with extubation was made by a doctor in the cardiothoracic surgical care unit who was blinded to the group allocation.

### 2.5 Study protocol: PCV-VG group

After tracheal intubation, mechanical ventilation started with a tidal volume (VT) of 8 mL/kg (using ideal body weight) with positive end-expiratory pressure (PEEP) at 5 cm H_2_O in the PCV-VG group. The set fraction of inspiration oxygen (FiO_2_) was 60%, the oxygen flow was 2–3 L/min, the initial inspiratory/expiratory (I: E) ratio was set to 1:2, and respiratory rate (RR) was adjusted to maintain an end-tidal carbon dioxide (ETCO2) concentration to 4.7–6.7 kPa.

Upon OLV started, the tidal volume was turn to 6 mL/kg with PEEP at 5 cm H_2_O. The set FiO_2_ was kept at 60%, the oxygen flow was 2–3 L/min, the I: E ratio was set to 1:2.5, and RR was adjusted to maintain an ETCO_2_ concentration to 4.7–6.7 kPa.

### 2.6 Study protocol: VCV group

Unlike the PCV-VG group, the mechanical ventilation mode was set to VCV mode after tracheal intubation in the VCV group. The parameters applied during TLV were: tidal volume 8 mL/kg; PEEP 5 cm H_2_O; FiO2 60%; oxygen flow 2–3 L/min; the I: E ratio 1:2, and RR adjusted to maintain ETCO_2_ 4.7–6.7 kPa.

The mechanical ventilation mode of VCV group remained VCV mode during OLV, and the ventilation parameter settings were the same as those of PCV-VG group. The values applied during OLV were: tidal volume 6 mL/kg; PEEP 5 cm H_2_O; oxygen flow 2–3 L/min; FiO_2_ 60%; the I: E ratio 1:2.5, and RR adjusted to maintain ETCO_2_ 4.7–6.7 kPa.

### 2.7 Lung protective ventilation strategy

Lung protective ventilation strategy was applied between the two groups. Lung recruitment was performed three times in both of the groups: after tracheal intubation, after initiate of OLV, and at the end of OLV (i.e., before two-lung ventilation was restarted). According to previous studies (1, 2), lung recruitment in this study was performed as follows: continuous airway pressure of 30 cmH_2_O, lasting 15–20s.

### 2.8 Outcome measures

#### 2.8.1 Data collection

Data on participants’ vital signs, artery blood gas analysis, and respiratory parameters were recorded at 5 important epochs: after anesthesia induction (T0), at the beginning of surgery (T1), 30 min after OLV (T2), at the end of OLV (T3), and at the end of surgery (T4). The Qs/Qt, oxygen saturation of blood (SpO_2_), PaO_2_, PaO_2_/FiO_2_ ratio, oxygenation index (OI; [FiO_2_ × mean airway pressure × 100]/PaO_2_) and oxygen saturation index (OSI; [FiO_2_ × mean airway pressure × 100]/SpO_2_) (3, 4) were calculated. Lung ultrasound (LUS) examination was performed at T0 and T4 and the results was recorded.

#### 2.8.2 Primary outcome

The primary outcome was the Qs/Qt, a key parameter reflecting the extent of intrapulmonary shunting and gas exchange impairment. Qs/Qt values were automatically derived at five critical time points using the cobas b 123 blood gas analyzers (Roche Diagnostics GmbH, Sandhofer Strasse 116, 68,305 Mannheim, Germany). These values were estimated based on the Berggren equation (4), utilizing the measurements obtained by the analyzer.

#### 2.8.3 Secondary outcomes

Secondary outcomes encompassed intraoperative respiratory parameters, including peak airway pressure (Ppeak), mean airway pressure, dynamic compliance, and airway resistance. Additionally, it included results of blood gas analysis, vital signs, findings from LUS examinations, postoperative mechanical ventilation duration (defined as the time in hours from surgery completion to extubation), and postoperative hospital stay (defined as the number of calendar days from surgery completion to readiness for hospital discharge). The occurrence of hypoxemia during OLV, defined as SpO_2_ < 92% or PaO_2_ < 8 kPa without any evidence of hemodynamic instability or airway compromise, was also recorded. In this trial, PPCs were defined as pneumonia, pneumothorax, pleural effusion, and atelectasis according to previous articles and clinical practice in our institution. Pneumonia was diagnosed using a combination of symptoms, laboratory test results, physical chest examinations, and chest radiography, following the European Perioperative Clinical Outcome (EPCO) criteria (5). Pleural effusion, atelectasis, and pneumothorax were identified based on chest radiography findings (5).

Two LUS examinations were conducted for all participants: one after anesthesia induction (T0) and another at the end of surgery (T4). The same experienced anesthesiologist, blinded to the group allocation, performed all LUS examinations. The LUS scans were performed using a SONIMAGE HS1 PLUS machine (KONICA MINOLTA, Tokyo, Japan) with a 4–18 MHz linear transducer. The participants were in the supine position during the lung scan. In accordance with article reported by Acosta et al. (6), each hemithorax was subdivided into six sections using three longitudinal lines (parasternal, anterior, and posterior axillary) and two axes (one above the diaphragm and the other 1 cm above the nipples). Following the approach outlined by Song et al. (7), the degree of juxtapleural consolidation and B-lines in each region were individually scored. The extent of juxtapleural consolidation was categorized into four levels and scored between 0 and 3: (0) no consolidation; (1) minimal juxtapleural consolidation; (2) small-sized consolidation; and (3) large-sized consolidation. Besides, the degree of B-lines was also scored between 0 and 3 as shown follow: (0) fewer than three isolated B-lines; (1) multiple well-defined B-lines; (2) multiple coalescent B-lines; and (3) white lung.

### 2.9 Sample size estimation

The required sample size was calculated by Power Analysis and Sample Size 15.0.5 (NCSS statistical software; NCSS LLC, Kaysville, UT, USA). We chose two-sample t-tests assuming unequal variance to calculate the sample size and used the following settings: *α* = 0.05, a power of 80%, two-sided, and group allocatio*n* = 1:1. According to the results of preliminary experiment, we calculated that 33 patients were needed for each of the two groups. Considering a 20% dropout rate, the total sample size was increased to 80 patients in the present study.

### 2.10 Statistical analysis

Statistical analyses were conducted using SPSS 25.0 (SPSS Inc., Chicago, IL, USA). The normality of continuous data was evaluated by the Kolmogorov–Smirnov test. Continuous variables are reported as either mean ± standard deviation (SD) (e.g., mean arterial pressure, hemoglobin) or median (interquartile range, IQR) (e.g., age, Qs/Qt, Ppeak). Categorical variables were represented as numbers (percentages). Student’s t-test was applied to assess group differences in normally distributed continuous data. While the Mann–Whitney U test was employed for comparing non-normally distributed continuous variables. And for categorical data with small cell counts, we computed the theoretical frequency. If the theoretical frequency was ≥5, we employed the χ^2^ test without Yates’ correction. If the theoretical frequency was <1, we utilized Fisher’s exact test. When the theoretical frequency fell between these two values, we applied the χ^2^ test with Yates’ correction. Adjusted logistic regression analysis was employed to compare categorical data, and the results were presented as odds ratio (OR) with 95% confidence interval (CI). Demographic characteristics such as age, sex, weight, and height were considered in the adjustment. For continuous variables (whether normally distributed or not), we computed the 95% CI around the difference to clarify confidence about the inferred effect size within the pediatric population. Among them, for continuous variables that did not follow a normal distribution, we employed the Hodges–Lehmann estimate to calculate the difference along with its 95%CI.

All hypothesis tests were two-sided, and *p* value <0.05 was considered statistically significant.

## 3 Results

### 3.1 Patient characteristics

**Figure 1:**
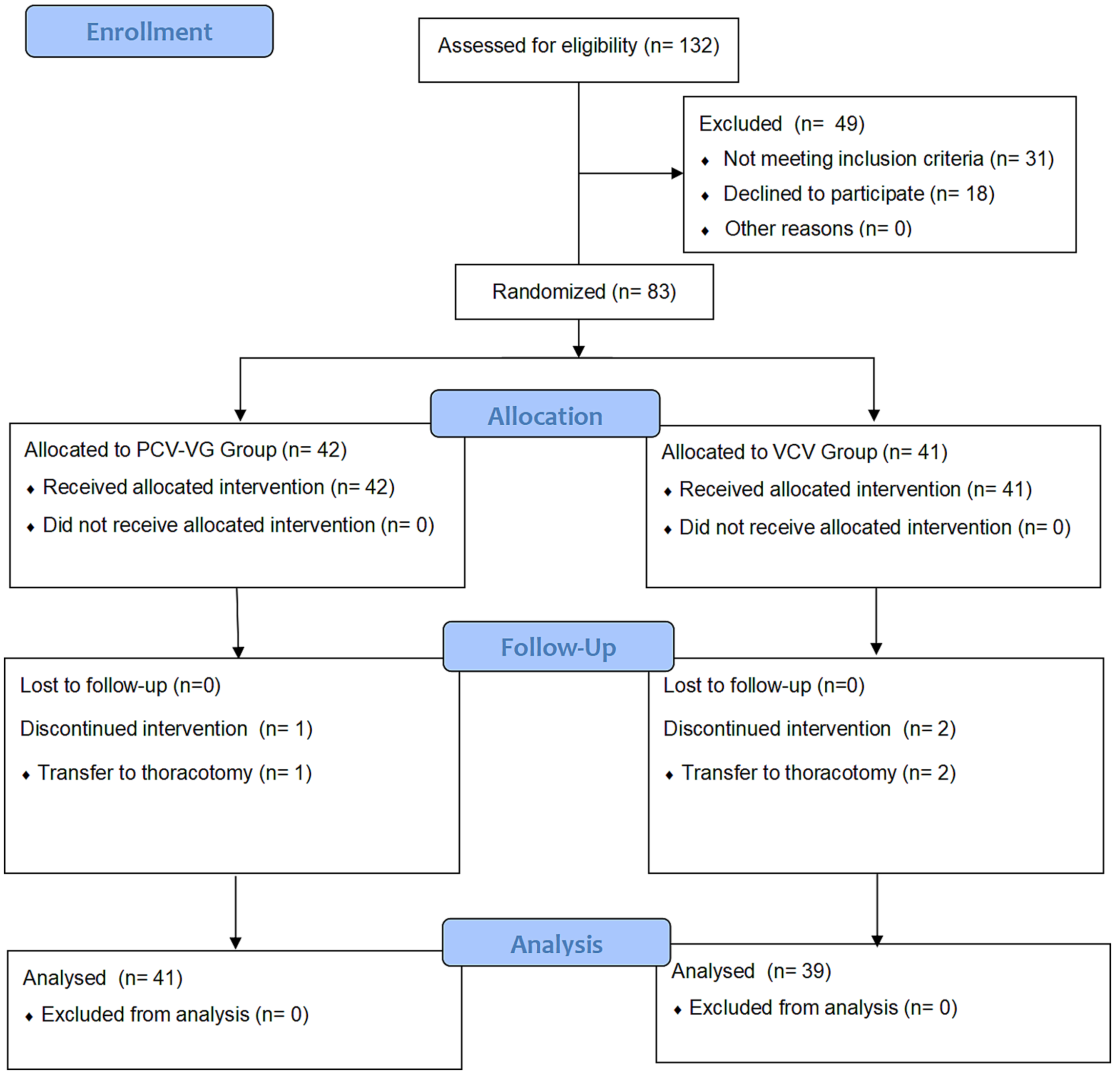
CONSORT flow diagram of patients’distribution. PCV-VG: pressure-controlled volume guaranteed ventilation; VCV, volume control ventilation. A total of 132 children who underwent thoracic surgery were evaluated for eligibility before enrollment. Forty-nine children were excluded from this study for not meeting the inclusion criteria or refusing to participate. Hence, 83 children were enrolled. After informed consent was obtained, eligible patients were prospectively randomized into either the PCV-VG group or the VCV group. Two patients in the PCV-VG group and 3 patients in VCV group discontinued intervention. Data from 41 children (PCV-VG group) and 39 children (VCV group) were included in the final analysis, respectively.

A total of 132 children were screened for eligibility, of whom 83 were enrolled and randomized between November 2022 and August 2023. One child in the PCV-VG group and two in the VCV group were excluded due to intraoperative conversion to thoracotomy. Consequently, data from 80 children (41 in the PCV-VG group and 39 in the VCV group) were analyzed according to intention-to-treat analysis, as illustrated in Figure 1.

Table 1 summarizes the baseline demographic characteristics and intraoperative variables. None of the children in either group required oxygen supplementation before surgery. All children underwent video-assisted thoracoscopic lobectomy for pulmonary sequestration (PS) or congenital cystic adenomatoid malformation (CCAM) performed by the same experienced surgical team.

**Table 1:** Baseline characteristics and intraoperative variables of children undergoing thoracic surgery requiring one-lung ventilation.

Parameters	PCV-VG group (*n* = 41)	VCV group (*n* = 39)	*p*-value
Age (yr)	2.8 (1.2–5.1)	2.7 (1.0–5.0)	0.784
Weight (kg)	13.0 (10.0–18.0)	12.3 (8.1–18.3)	0.992
Height (cm)	99.0 (84.0–111.0)	100.0 (79.0–117.0)	0.965
Sex (male)	22 (53.7%)	22 (56.4%)	0.826
ASA physical status			0.433
I-II	36 (87.8%)	37 (94.9%)	
III	5 (12.2%)	2 (5.1%)	
Type of disease			0.641
CCAM	26 (63.4%)	27 (69.2%)	
Lung isolation	15 (36.6%)	12 (30.8%)	
Side of OLV (left/right)			0.655
Left	22 (53.7%)	18 (46.2%)	
Right	19 (46.3%)	21 (53.8%)	
Placement method of bronchial blocker			> 0.999
Intraluminal	13 (31.7%)	12 (30.8%)	
Extraluminal	28 (68.3%)	27 (69.2%)	
Duration of anesthesia (min)	200 (160, 250)	225 (150,270)	0.722
Duration of surgery (min)	123 (81, 175)	142 (90, 185)	0.482
Duration of OLV (min)	75 (48, 136)	80 (45, 142)	0.908
Total infusion fluid (ml)	450 (275, 415)	430 (300, 600)	0.798
Urine volume (ml)	100 (60, 200)	150 (50, 240)	0.768
Blood loss (ml)	15 (10, 30)	20 (10, 50)	0.127
Allogeneic blood transfusion^a^	2 (4.9%)	3 (7.5%)	0.676

Data are presented as mean (standard deviation), median (inter-quartile range), or *n* (%). PCV-VG: pressure-controlled volume guaranteed ventilation; VCV, volume control ventilation; ASA, American society of aneshesiologists; CCAM, congenital cystic adenomatoid malformation of lung; PS, pulmonary sequestration; OLV, one-lung ventilation.

^a^ Two people in the PCV-VG group needed blood transfusion, 160 mL and 320 mL respectively, and three people in the VCV group needed blood transfusion, 160 mL, 200 mL and 240 mL, respectively.

### 3.2 Primary outcome

When comparing TLV to OLV, both groups of children exhibited an elevated Qs/Qt ratio during OLV. Notably, the VCV group demonstrated a significantly higher Qs/Qt ratio than the PCV-VG group both at 30 min after OLV (T2) and at the end of OLV (T3) (all *p* < 0.0001). Following the transition from OLV to TLV, both groups exhibited a declining Qs/Qt trend. However, at the end of surgery (T4), the PCV-VG group displayed a lower Qs/Qt (*p* = 0.012) (Table 2). Through further age-cohorted analysis, we found that the difference in Qs/Qt is manifested in the age range of 1–6 years old (Supplementary Table 2).

**Table 2:** Primary and secondary outcomes for children allocated randomly to PCV-VG group or VCV group.

Parameters	PCV-VG Group (*n* = 41)	VCV Group (*n* = 39)	Difference (95%CI)	*P -*value
Primary outcome-Qs/Qt				
T0	6.3 (5.4, 8.0)	6.0 (4.7, 8.6)	−0.27 (−1.1–0.74)	0.532
T1	7.3 (6.4, 8.6)	7.6 (6.8, 10.5)	0.46 (−0.31–1.33)	0.275
T2	12.7 (10.4, 15.9)	18.2 (15.3, 20.2)	4.78 (3.35–6.34)	< 0.0001
T3	9.1 (8.2, 10.9)	11.4 (9.7, 14.6)	2.06 (1.03–3.39)	< 0.0001
T4	7.1 (6.1, 8.5)	8.1 (7.2, 10.4)	1.12 (0.22–2.07)	0.012
Secondary outcomes				
PPCs^a^	3 (7.3%)	15 (38.5%)		0.001
Respiratory infection	1 (2.4%)	5 (12.8%)		0.104
Atelectasis	0	4 (10.3%)		0.052
Respiratory failure	0	0		
Pleural effusion	0	4 (10.3%)		0.052
Pneumothorax	2 (4.9%)	7 (18.0%)		0.084
Bronchospasm	0	0		
Hypoxemia	0	3 (7.5%)		0.116
Lowest oxygen saturation (%)	98 (96, 100)	94 (92, 96)	−4 (−5 - −3)	<0.0001
Duration of postoperative mechanical ventilation (min)	110 (70, 140)	120 (100, 140)	20 (0–40)	0.036
Postoperative hospital stays (day)	7 (5, 9)	8 (7, 15)	1 (0–4)	0.054

Data are presented as mean (standard deviation), median (inter-quartile range), or *n* (%). PCV-VG, pressure-controlled volume guaranteed ventilation; VCV, volume control ventilation; Qs/Qt, intrapulmonary shunt rate; PPCs, postoperative pulmonary complications.

^a^adjusted odds ratio (95% CI): 0.13 (0.03–0.50).

T0, after anesthesia induction; T1, at the beginning of surgery; T2, 30 min after OLV; T3, at the end of OLV; T4, at the end of surgery.

### 3.3 Secondary outcomes

**Figure 2:**
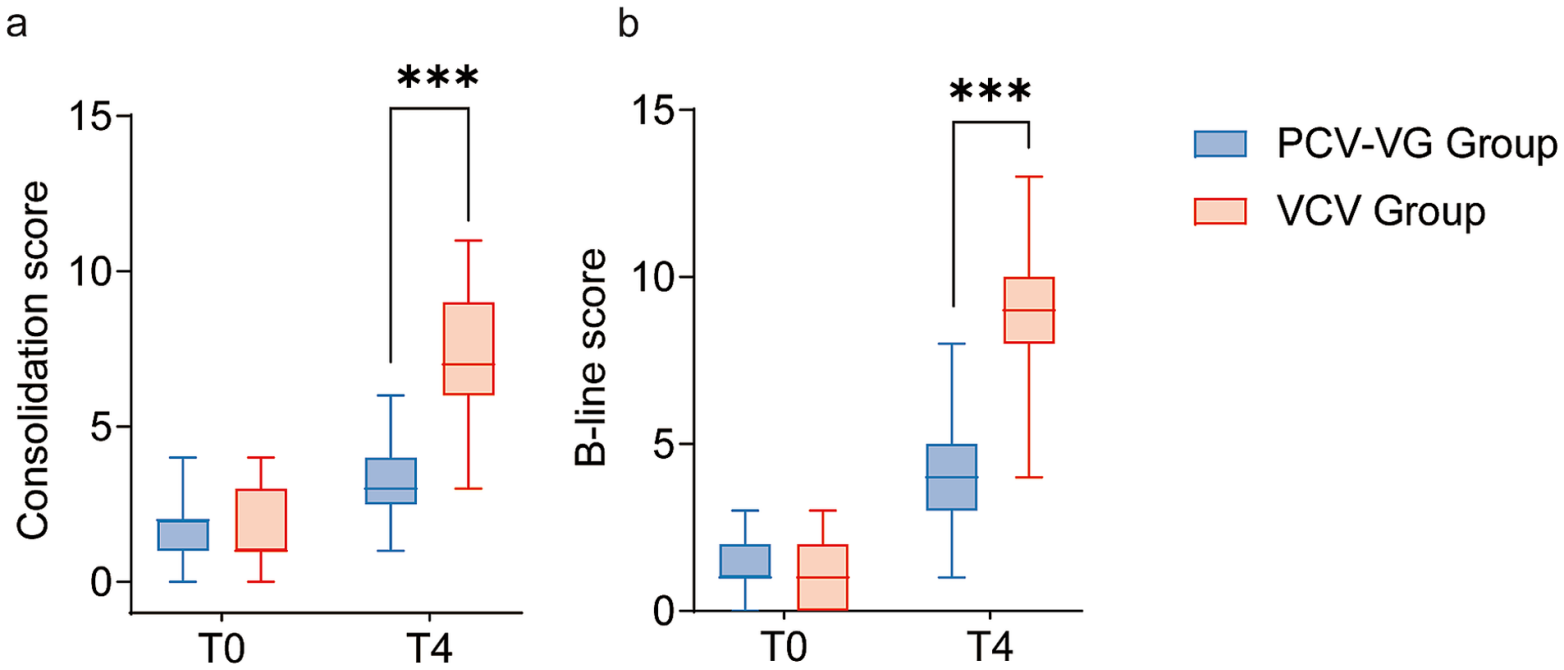
The Consolidation scores **(a)** and B-line scores **(b)** for children receiving the volume control ventilation mode (VCV group, red box) or the pressure-controlled volume guaranteed ventilation mode (PCV-VG group, blue box). The bold black line represents the median, the ends of the boxes indicate interquartile ranges and error bars indicate 10 and 90 percentiles.

One child in the PCV-VG group and two in the VCV group (*p* = 0.611) experienced blocker dislodgement during the trial, which was resolved after fiberoptic bronchoscopy and readjustment. No other intraoperative complications were noted. Within 72 h of surgery, PPCs occurred in 3 (7.3%) children in the PCV-VG group and 15 (38.5%) children in the VCV group (adjusted OR 0.13, 95%CI 0.03–0.50, *p* = 0.001). No differences were found among the individual components of the pulmonary complication composite outcome (Table 2). Regarding hypoxemia, even though there was a significant difference in SpO_2_ during OLV between the two groups, there was no statistically significant difference in the incidence of hypoxemia (Table 2 and Supplementary Table 1). Subsequent analysis indicated that the median lowest SpO2 during surgery in the VCV group (94%) was markedly lower than that in the PCV-VG group (98%) (difference, −4%; 95% CI, −5% to −3%, *p* < 0.0001, as presented in Table 2). The mean duration of postoperative mechanical ventilation in the PCV-VG group (110 min) was significantly shorter than that in the control group (120 min) (difference, 20; 95% CI, 0 to 40, *p* = 0.036) (Table 2). Regarding the results of LUS assessment, the median LUS scores at the end of surgery (T4) were significantly lower in the PCV-VG group than that in the VCV group in terms of consolidation score [3 (3, 4) vs. 9 (7, 10); *p* < 0.0001] and B-lines score [4 (3, 5) vs. 9 (8, 10); *p* < 0.0001] (shown in Figure 2).

Table 3 summarized intraoperative respiratory parameters and certain outcomes calculated from these parameters. The tidal volume was similar between the groups, both during two-lung ventilation and one-lung ventilation. From T2 to T4, the Ppeak in the PCV-VG group were lower than those in the VCV group. At T2, the highest perioperative airway pressure was observed. At this time, the median Ppeak in the PCV-VG group was 20 cmH_2_O, which was significantly lower than that in the VCV group [25 cmH_2_O, difference (95% CI), 5 (4 to 6), *p* < 0.0001]. Concerning the results of airway resistance (Raw), it was observed that during OLV, the results of Raw in the PCV-VG group were lower than those of the VCV group. However, these differences reached statistical significance only at T2 [difference (95%CI), 11.5 (6.2 to 16.8), *p* = 0.047]. Based on the formula calculation, it was determined that the experimental group using the PCV-VG ventilation mode exhibited slightly improved dynamic lung compliance (Cydn) during OLV compared to the control group using the VCV ventilation mode (*p* = 0.015 at T2 and *p* = 0.037 at T3). The OI was significantly higher in the VCV group at both T2 and T3 than in the PCV-VG group (*p* = 0.001 at T2 and *p* = 0.006 at T3). Similarly, comparable trends were observed in the OSI at T2 and T3 (*p* = 0.005 and *p* = 0.001, respectively).

**Table 3:** Intraoperative respiratory parameters.

Parameters	PCV-VG group (*n* = 41)	VCV group (*n* = 39)	Difference (95%CI)	*P*-value
Tidal volume (ml)				
T0	115 (84, 149)	118 (85, 152)	0 (−18–24)	0.897
T1	116 (88, 152)	118 (85, 152)	0 (−20–22)	0.973
T2	87 (66, 114)	90 (65,114)	0 (−15–17)	0.996
T3	87 (65, 114)	90 (64, 114)	0 (−15–16)	0.988
T4	116 (88, 147)	120 (85, 152)	0 (−19–23)	0.923
Peak airway pressure (cmH_2_O)				
T0	14 (12, 15.5)	15 (14, 16)	1 (0–2)	0.082
T1	15 (14, 17)	17 (16, 18)	1 (0–2)	0.060
T2	20 (18.5, 22)	25 (23, 27)	5 (4–6)	< 0.0001
T3	17 (15.5, 18)	20 (19, 23)	4 (2–5)	< 0.0001
T4	15 (13, 16)	18 (15, 19)	2 (1–3)	0.001
Plateau pressure (cmH_2_O)				
T0	14 (12, 15)	14 (13, 16)	1 (0–2)	0.203
T1	14 (14, 16.5)	16 (15, 17)	1 (0–2)	0.091
T2	20 (18, 21)	24 (22, 26)	4 (3–5)	< 0.0001
T3	16 (15, 18.5)	20 (18, 23)	4 (2–5)	< 0.0001
T4	15 (13, 16)	16 (14, 18)	2 (1–3)	0.003
Raw				
T0	36.2 ± 9.4	37.4 ± 10.6	1.2 (−3.2–5.7)	0.931
T1	39.2 ± 9.4	41.6 ± 9.5	2.3 (−1.9–6.5)	0.755
T2	49.6 ± 10.4	61.1 ± 13.1	11.5 (6.2–16.8)	0.047
T3	37.6 ± 8.4	46.0 ± 10.2	8.4 (4.2–12.5)	0.062
T4	30.3 ± 5.6	37.4 ± 7.8	7.2 (4.1–10.2)	0.072
Cydn (ml/cmH_2_0)				
T0	13 (10–18.5)	8 (12–17)	−1 (−4–2)	0.412
T1	11 (8.5–14.5)	11 (7–15)	−1 (−3–1)	0.505
T2	6 (4–8)	5 (3–6)	−1 (−2–0)	0.015
T3	7 (5–10.5)	6 (4–8)	−1 (−3–0)	0.037
T4	11 (9–16.5)	10 (7–14)	−2 (−4–1)	0.132
OI				
T0	1.7 (1.4, 1.9)	1.7 (1.5, 2.1)	0.11 (−0.07–0.28)	0.333
T1	2.0 (1.7, 2.3)	2.1 (1.7, 2,4)	0.10 (−0.08–0.29)	0.227
T2	4.2 (3.5, 4.8)	5.0 (4.0, 6.2)	1.06 (0.04–1.62)	0.001
T3	2.3 (2.1, 2.7)	2.7 (2.3, 3.4)	0.39 (0.12–0.69)	0.006
T4	1.7 (1.5, 2.0)	1.7 (1.5, 2.0)	−0.03 (−1.18–0.12)	0.617
OSI				
T0	4.2 (3.6, 4.2)	4.2 (3.6, 4.8)	0.00 (0.00–0.60)	0.234
T1	4.2 (3.7, 4.8)	4.3 (4.2, 4.9)	0.19 (−0.04–0.55)	0.275
T2	5.4 (5.0, 5.8)	5.9 (5.2, 6.5)	0.46 (0.12–0.79)	0.005
T3	4.2 (4.2, 4.8)	4.8 (4.3, 5.1)	0.27 (0.10–0.67)	0.001
T4	3.6 (3.6, 4.2)	4.2 (3.6, 4.2)	0.00 (−0.04–0.11)	0.608

Data are presented as mean (standard deviation), median (inter-quartile range), or *n* (%). PCV-VG, pressure-controlled volume guaranteed ventilation; VCV, volume control ventilation; CI, confidence interval.

T0, after anesthesia induction; T1, at the beginning of surgery; T2, 30 min after OLV; T3, at the end of OLV; T4, at the end of surgery.

Table 4 summarizes the key results of blood gas analysis for both groups. Within each group, the PaO_2_ during OLV was lower than during TLV, whereas the trend for PaCO_2_ was the opposite. At T2 and T3, the PCV-VG group exhibited higher PaO_2_ values compared to the VCV group, with concomitantly lower PaCO_2_ levels. The PaO_2_/FiO_2_ of both groups during OLV exhibited a decreasing trend compared to TLV. Additionally, in the VCV group, the PaO_2_/FiO_2_ at T3 and T4 was lower than that in the PCV-VG group (*p* = 0.002 and *p* = 0.028, respectively). The trend of changes in a/AO_2_ remained consistent between the two groups, and at T2 and T3, the a/AO_2_ in the PCV-VG group were higher than those in the VCV group, although the difference was not statistically significant. Both groups experienced respiratory index (RI) > 100% during OLV, with the median RI in the VCV group (270% at T2, 160% at T3) being higher than that in the PCV-VG group (148% at T2, 93% at T3) (all *p* < 0.001).

**Table 4:** Results of intraoperative arterial blood gas analysis.

Parameters	PCV-VG group (*n* = 41)	VCV group (*n* = 39)	Difference (95%CI)	*P*-value
PaO2 (mmHg)				
T0	238.0 (212.2, 283.7)	229.8 (208.8, 272.0)	−6.60 (−25.00–10.4)	0.456
T1	214.5 (203.8, 240.2)	208.3 (195.4, 241.9)	−9.50 (−22.30–6.10)	0.225
T2	129.2 (115.3, 155.6)	105.7 (89.4, 137.5)	−21.00 (−33.50- -7.60)	0.002
T3	189.5 (168.3, 197.8)	176.8 (143.9, 191.1)	−13.80 (−27.70- -2.00)	0.028
T4	226.0 (207.4, 239.0)	227.7 (211.3, 254.8)	5 (−8.50–18.9)	0.416
PaCO2 (mmHg)				
T0	37.9 ± 3.1	37.1 ± 3.0	0.85 (−0.51–2.21)	0.868
T1	38.9 ± 2.9	39.2 ± 3.9	−0.27 (−1.80–1.25)	0.205
T2	42.8 ± 2.8	48.1 ± 4.1	−5.32 (−6.89- -3.75)	0.040
T3	40.1 ± 3.4	43.2 ± 4.9	−2.53 (−4.42- -0.63)	0.026
T4	39.2 ± 2.1	39.2 ± 2.8	−0.04 (−1.13–1.06)	0.062
PaO2/FiO2 (mmHg)				
T0	396.7 (353.6, 472.8)	383.0 (348.0, 453.3)	−11.0 (−41.7–17.3)	0.456
T1	357.5 (339.6, 400.4)	347.2 (325.7, 403.2)	−15.8 (−37.1–10.2)	0.225
T2	215.3 (192.1, 259.3)	176.2 (149.0, 229.2)	−35.1 (−55.9- −12.7)	0.002
T3	315.8 (280.4, 329.6)	294.7 (239.8, 318.5)	−23.0 (−46.1- −3.3)	0.028
T4	376.7 (345.6, 398.3)	379.5 (352.2, 424.7)	8.4 (−14.2–31.5)	0.416
a/AO2				
T0	59.7 ± 10.8	57.1 ± 23.7	2.6 (−2.8–8.1)	0.182
T1	55.6 ± 13.2	51.2 ± 12.5	4.4 (−1.3–10.1)	0.687
T2	39.5 ± 11.3	26.8 ± 9.4	12.7 (8.1–17.3)	0.107
T3	49.8 ± 12.0	40.9 ± 14.2	9.0 (3.1–14.9)	0.090
T4	61.1 ± 10.7	59.8 ± 11.1	1.3 (−3.6–6.1)	0.710
RI (%)				
T0	64 (36.5, 76)	50 (37, 79)	−2 (−13–7)	0.718
T1	70 (44.5, 80)	73 (53, 97)	9 (−4–22)	0.154
T2	148 (116, 203)	270 (213, 287)	98 (69–128)	< 0.0001
T3	93 (70.5, 105.5)	160 (81, 192)	57 (24–80)	< 0.0001
T4	61 (45, 74)	75 (49, 94)	13 (0–25)	0.058

Data are presented as mean (standard deviation), median (inter-quartile range), or n (%). PCV-VG, pressure-controlled volume guaranteed ventilation; VCV, volume control ventilation; CI, confidence interval.

T0, after anesthesia induction; T1, at the beginning of surgery; T2, 30 min after OLV; T3, at the end of OLV; T4, at the end of surgery.

## 4 Discussion

In this randomized controlled study, children in the PCV-VG group had significantly lower Qs/Qt than those in the VCV group during OLV. Moreover, the application of PCV-VG correlated with improved intraoperative oxygenation, lower airway pressure, reduced lung atelectasis and PPCs, and shorter postoperative mechanical ventilation duration.

Different ventilation modes have different working principles. VCV, being the most classic ventilation mode during pediatric OLV, can deliver a constant tidal volume during both OLV and TLV, but it also elevates the risk of barotrauma (8). PCV-VG amalgamates the characteristics of VCV and pressure-controlled ventilation, utilizes a decelerated flow pattern to deliver a constant tidal volume at the lowest inspiratory airway pressure (9). However, clinical data on the application of PCV-VG in children with OLV are scarce. To our knowledge, this is the first RCT investigating the impact of ventilation mode on children undergoing thoracic surgery, with the primary outcome being Qs/Qt. Owing to factors like lateral positioning and specific physiological mechanisms in children (e.g., a less rigid cartilaginous rib cage, excessive pressure transmitted through the diaphragm, and a higher metabolic rate), children are more prone to experiencing substantial reductions in ventilation/perfusion (V/Q) ratio and increasing Qs/Qt compared to adults, thus predisposing them to hypoxemia (10, 11). Our study demonstrated that, in comparison to VCV, PCV-VG effectively enhances Qs/Qt during OLV. Simultaneously, we observed higher PaO_2_/FiO_2_ and RI in the PCV-VG group, indirectly confirming the improvement of V/Q matching by PCV-VG. Despite there were statistical differences between groups for OI and OSI during OLV, all values remained within the mild acute respiratory distress syndrome range.

Given the disparities in respiratory physiology between children and adults, avoiding high tidal volumes and minimizing the risk of barotrauma is considered prudent (12, 13). Our study revealed that the Ppeak was lower in patients assigned to the PCV-VG group compared to the VCV group, aligning with findings from previous studies on ventilation mode types (14–16). Respiratory resistance in the PCV-VG group remained lower than that in the VCV group throughout OLV. Additionally, dynamic compliance was higher in children allocated to the PCV-VG group compared to the VCV group. Therefore, similar to other clinical studies on thoracic surgery (17–19), our research further supports the significant benefits of using PCV-VG mode during pediatric OLV, resulting in reduced airway pressure and improved airway dynamics.

OLV causing surgical lung collapse and imbalanced V/Q matching, ultimately leading to varying degrees of lung injury (11, 20). Clinical research evidence suggests that VILI could worsen existing lung injury or render the lungs more susceptible to further injury. In other words, this phenomenon is described as the “two-hit lung injury model,” and it is significantly associated with massive blood transfusions, sepsis, cardiopulmonary bypass, and pulmonary ischemia–reperfusion injury (21, 22). In this prospective study, children who applied PCV-VG mode exhibited lower Qs/Qt and Ppeak, undoubtedly minimizing VILI. Simultaneously, the results regarding the atelectasis score at the end of surgery and the incidence of PPCs within 72 h after surgery can also signify the alleviating impact of employing PCV-VG on ventilator-induced lung injury from a clinical prognosis perspective. Some studies suggest that the choice of ventilation mode during OLV does not mitigate lung injury and does not improve patient prognosis (14, 23, 24). This contrasts with our research findings, which may be attributed to differences in the study population (adults *vs.* children), surgical trauma/manipulation, OLV duration, and some studies not incorporating PCV-VG patterns.

Similar to other previous RCTs investigating the optimal ventilation protocol and lung protection during pediatric surgery (13, 25), although many clinically significant indicators (such as LUS results, incidence of PPCs, duration of postoperative mechanical ventilation) exhibit statistically significant differences, these differences appear relatively small compared to adult thoracic surgeries that require OLV. Possible reasons for this phenomenon include the predominance of congenital pulmonary malformation among children, generally better pulmonary conditions, and the absence of other systemic diseases present before surgery.

Due to the distinctive characteristics of the pediatric population, there is limited access to high-quality research and literature regarding the application of various ventilation modes in children undergoing OLV. Based on these knowledge gaps, we designed this study and confirmed the safety and benefits of applying PCV-VG mode in children undergoing OLV, but there are still several limitations to this study that need to be considered. Firstly, inflammatory biomarkers and oxidative stress indexes were not measured for the direct evaluation of lung injury. Secondly, this study concentrated on the immediate occurrence of lung injury (findings from LUS examinations) and in the short term (the incidence of PPCs), but did not explore long-term lung injury after surgery. Thirdly, although we found that the application of PCV-VG was more effective in pre-school children aged 1–6 through age grouping, this conclusion cannot be generalized due to the small sample size in each group after grouping. Further research with larger sample sizes is needed to validate this finding.

In summary, this RCT supports the significant benefits of applying the PCV-VG mode in children undergoing video-assisted thoracoscopic lobectomy. Utilizing the PCV-VG mode in children with OLV can effectively improve Qs/Qt during OLV, elevate oxygenation, decrease airway pressure, improved airway dynamics, mitigate VILI, and lower the incidence of PPCs. Our findings could be extended to the anesthesia approaches in pediatric lobectomy with OLV.

## Data Availability

Publicly available datasets were analyzed in this study. This data can be found here: the data that support the findings of this study are available from the corresponding author Shuangquan Qu upon reasonable request.
