# Supplementary material for: Effects of pressure-controlled ventilation-volume guaranteed on children undergoing thoracic surgery: a prospective, randomized controlled trial
Source: Front Med (Lausanne). 2025 Sep 15;12:1647682. doi: 10.3389/fmed.2025.1647682 (PMC12477203; doi:10.3389/fmed.2025.1647682)
Supplement: Supplementary file 1 [file Table_1.DOCX]

**Supplementary Table 1** Part results of arterial blood gas analysis and intraoperative monitoring data. Data are presented as mean (standard deviation), median (inter-quartile range), or n (%). PCV-VG: pressure-controlled volume guaranteed ventilation; VCV: volume control ventilation; CI: confidence interval;

T0: after anesthesia induction; T1: at the beginning of surgery; T2: 30 minutes after OLV; T3: at the end of OLV; T4: at the end of surgery.

|  | PCV-VG Group  (n= 41) | VCV Group  (n= 39) | *P*-value |
| --- | --- | --- | --- |
| PH |  |  |  |
| T0 | 7.38±0.03 | 7.39±0.03 | 0.631 |
| T1 | 7.37±0.03 | 7.38±0.04 | 0.289 |
| T2 | 7.32±0.05 | 7.29±0.04 | 0.736 |
| T3 | 7.34±0.04 | 7.34±0.05 | 0.454 |
| T4 | 7.37±0.03 | 7.36±0.03 | 0.767 |
| Glu |  |  |  |
| T0 | 4.7±0.5 | 4.8±0.6 | 0.092 |
| T1 | 4.7±0.5 | 4.7±0.6 | 0.237 |
| T2 | 5.2±0.7 | 5.4±0.7 | 0.870 |
| T3 | 5.2±0.7 | 5.4±0.7 | 0.930 |
| T4 | 5.2±0.8 | 5.4±0.7 | 0.440 |
| Hb |  |  |  |
| T0 | 10.5±1.3 | 10.5±1.3 | 0.915 |
| T1 | 10.3±1.0 | 10.3±1.1 | 0.664 |
| T2 | 10.3±1.1 | 10.3±1.1 | 0.716 |
| T3 | 10.3±1.1 | 10.1±1.2 | 0.849 |
| T4 | 10.2±1.2 | 10.1±1.1 | 0.780 |
| MAP (mmHg) |  |  |  |
| T0 | 61.9±9.0 | 62.0±9.7 | 0.801 |
| T1 | 60.6±8.2 | 60.8±10.3 | 0.372 |
| T2 | 56.8±7.0 | 56.5±8.2 | 0.254 |
| T3 | 58.5±7.1 | 56.5±8.0 | 0.938 |
| T4 | 59.4±7.1 | 58.4±7.7 | 0.986 |
| HR |  |  |  |
| T0 | 109.3±12.5 | 114.0±15.6 | 0.305 |
| T1 | 102.1±12.6 | 106.3±13.4 | 0.983 |
| T2 | 105.2±12.1 | 109.0±13.3 | 0.608 |
| T3 | 106.4±11.9 | 109.9±13.9 | 0.390 |
| T4 | 105.8±11.9 | 109.9±14.3 | 0.180 |
| SpO2 (%) |  |  |  |
| T0 | 100(100, 100) | 100(100, 100) | 0.970 |
| T1 | 100(99, 100) | 100(98, 100) | 0.902 |
| T2 | 99(97, 100) | 95(92, 96) | < 0.0001 |
| T3 | 100(99, 100) | 97(95, 99) | < 0.0001 |
| T4 | 100(100, 100) | 100(99, 100) | 0.042 |

**Supplementary Table 2.** The primary outcome of two groups after age-cohorted.

|  | PCV-VG Group  (n= 41) | VCV Group  (n= 39) | *P*-value |
| --- | --- | --- | --- |
| T0 | | | |
| < 1 year old  (infant) | 6.5(4.8, 8.2) | 9.1(4.1, 11.6) | 0.643 |
| 1-6 years old  (Pre-school age) | 6.3(5.4, 7.5) | 5.8(4.7, 7.5) | 0.268 |
| 6-18 years old  (School age) | 6.4(5.6, 9.2) | 7.0(5.2, 8.8) | 0.738 |
| T1 | | | |
| < 1 year old  (infant) | 7.5(5.5, 9.5) | 9.3(6.3, 11.6) | 0.464 |
| 1-6 years old  (Pre-school age) | 7.2(6.2, 8.0) | 7.4(6.8, 8.0) | 0.562 |
| 6-18 years old  (School age) | 8.1(6.8, 10.3) | 8.2(6.5, 11.0) | 0.779 |
| T2 | | | |
| < 1 year old  (infant) | 10.2 (9.3, 11.1) | 18.8 (15.6, 20.6) | 0.071 |
| 1-6 years old  (Pre-school age) | 12.4 (10.4, 15.5) | 17.3 (14.9, 19.9) | <0.0001 |
| 6-18 years old  (School age) | 16.3 (11.7, 17.7) | 20.1 (17.3, 23.1) | 0.016 |
| T3 | | | |
| < 1 year old  (infant) | 8.4(7.8, 9.0) | 11.9(10.9, 13.7) | 0.071 |
| 1-6 years old  (Pre-school age) | 9.0(8.0, 9.9) | 10.6(9.7, 14.6) | 0.001 |
| 6-18 years old  (School age) | 10.9(8.9, 13.7) | 11.8(9.0, 15.4) | 0.366 |
| T4 | | | |
| < 1 year old  (infant) | 6.9(5.9, 7.9) | 9.1(6.8, 11.8) | 0.393 |
| 1-6 years old  (Pre-school age) | 6.5(5.9, 8.4) | 7.6(7.0, 10.2) | 0.029 |
| 6-18 years old  (School age) | 8.7(7.2, 9.5) | 8.6(6.9, 10.4) | >0.999 |
